# Effect of deep inspiration on impulse oscillometry responses to methacholine in healthy adults

**DOI:** 10.3389/falgy.2026.1816138

**Published:** 2026-06-01

**Authors:** Thomas Ringbaek, Tobias Folkmann, Soerine Neumann, Lars Frølund, Jann Mortensen, Charlotte S. Ulrik, Henrik H. El Ali

**Affiliations:** 1 Allergy and Lung Clinic, Helsingør, Denmark; 2 Department of Clinical Physiology and Nuclear Medicine, Copenhagen University Hospital—Rigshospitalet, Copenhagen, Denmark; 3 Department of Clinical Medicine, Faculty of Health and Medical Sciences, University of Copenhagen, Copenhagen, Denmark; 4 Department of Medicine, The National Hospital, University of the Faroe Islands, Torshavn, Faroe Islands; 5 Respiratory Research Unit Hvidovre, Department of Respiratory Medicine, Copenhagen University Hospital—Amager and Hvidovre Hospital, Hvidovre, Denmark; 6 Department of Biomedical Sciences, Copenhagen University, Copenhagen, Denmark

**Keywords:** airway hyperresponsiveness, asthma physiology, deep inspiration, impulse oscillometry, methacholine challenge test, respiratory mechanics, small-airway dysfunction

## Abstract

**Background:**

Impulse oscillometry (IOS) detects small-airway hyperresponsiveness during methacholine challenge testing (MCT). Deep inspiration (DI) may attenuate these responses, but the magnitude and interpretive impact of this effect in healthy adults remain uncertain.

**Methods:**

We conducted a prospective two-period crossover study in 38 healthy, non-smoking adults (median age 34.8 years; 53% female). Each participant completed two standardized five-dose MCT protocols: (i) IOS-only and (ii) IOS immediately followed by spirometry (IOS + DI). The primary endpoint was the within-subject difference in total resistance at 5 Hz (*Δ*R₅) at the maximum methacholine dose. Secondary analyses included R₅–R₂₀, AX, Fres, and bootstrap-derived upper limits of normal (ULNs).

**Results:**

No participant met the PD₂₀FEV₁ criterion. At the final dose, the IOS-only protocol elicited larger responses than IOS + DI (median *Δ*R₅% + 87% vs. +60%; paired difference +27%, 95% CI +12 to +44; *p* = 0.002). Similar attenuation was observed for R₅–R₂₀, AX, and Fres (all *p* ≤ 0.001), while central resistance (R₂₀) remained unaffected. Baseline R₅ correlated with *Δ*R₅ under both protocols (*ρ* ≈ 0.5, *p* < 0.01). Bootstrap-derived ULNs exceeded manufacturer thresholds, particularly under IOS-only conditions.

**Conclusion:**

Deep inspiration substantially attenuates methacholine-induced IOS responses, underscoring the need for protocol-specific diagnostic thresholds. IOS-only MCT provides sensitive, effort-independent information and may be particularly valuable when spirometry is impracticable.

**Clinical Trial Registration:** ClinicalTrials.gov NCT05012345*.*

## 1 Introduction

Asthma is a heterogeneous airway disease marked by variable symptoms, intermittent airflow limitation, and bronchial hyperresponsiveness (BHR) (1). The methacholine challenge test (MCT) remains the reference for quantifying BHR, typically defining a positive response as a ≥ 20% fall in FEV₁ (PD₂₀FEV₁) (2). Impulse oscillometry (IOS), performed during quiet tidal breathing, offers complementary, effort-independent indices that are especially sensitive to small-airway dysfunction, including total resistance at 5 Hz (R₅), central resistance at 20 Hz (R₂₀), frequency dependence of resistance (R₅–R₂₀), reactance at 5 Hz (X₅), resonant frequency (Fres), and reactance area (AX) (3–6). Reference equations derived from large population datasets confirm that these metrics are highly repeatable across visits (7, 8), supporting their use as objective endpoints when reproducible FEV₁ measurements cannot be obtained. Nonetheless, diagnostic cutoffs remain poorly standardized (9). This limitation is further compounded by the absence of universally accepted reference equations for IOS-derived bronchial responsiveness during methacholine challenge testing. For example, during MCT, a ≥ 20% decrease in FEV1 typically corresponds to a 40%–50% increase in R5 (6, 10), a threshold often suggested by the device manufacturers (11). However, IOS parameter thresholds must be established based on their ability to distinguish asthma from other lung diseases and healthy individuals, as well as to differentiate between well-controlled and uncontrolled asthma.

The IOS response to methacholine challenge in healthy adults has been investigated in a limited number of studies, either in conjunction with simultaneous spirometry (12–14) or without incorporating any deep inspiration (DI) maneuvers (14–18). However, these studies involved small sample sizes, not standardized MCT, or cohorts with potential confounding factors such as obesity or exposure to respiratory pollutants, which may influence airway impedance measurements. Assessing responses in rigorously screened, disease-free adults is therefore an essential first step toward meaningful interpretation of IOS-based MCT.

DI during MCT attenuates FEV₁ decline in both asthmatic and healthy individuals, while it provides a bronchoprotective effect only in healthy adults (19–22). Guidelines consequently recommend recording IOS before any forced maneuver at each dose step (5). The quantitative impact of DI on IOS during MCT has only recently been explored, notably by Henry et al. (14, 15). These studies compared single versus multiple methacholine doses in both healthy individuals and patients with asthma (15), as well as standardized MCT protocols with or without concurrent spirometry in asthmatic patients (14). As such, studying IOS responses during MCT in healthy individuals remains highly relevant, particularly for understanding the mechanisms underlying DI-induced bronchoprotection/dilation and for distinguishing physiological from pathological responses.

### 1.1 Objective

We aimed to quantify how a DI-requiring spirometry maneuver affects IOS responses during standard MCT in healthy adults and to derive protocol-specific upper limits of normal (ULN) for IOS outcomes to support clinical interpretation.

Here, we provide protocol-specific normative thresholds and quantify the cumulative bronchoprotective effect of deep inspiration during methacholine challenge testing in rigorously screened healthy adults.

## 2 Methods

### 2.1 Study design and oversight

We performed a single-center, prospective, two-period crossover study in healthy adults, registered at ClinicalTrials.gov (NCT05012345) (23) and conducted in accordance with CONSORT-Crossover guidance. The Regional Health Research Ethics Committee (Capital Region of Denmark) approved the protocol (H-25000081). All participants provided written informed consent.

### 2.2 Participants

**Figure 1:**
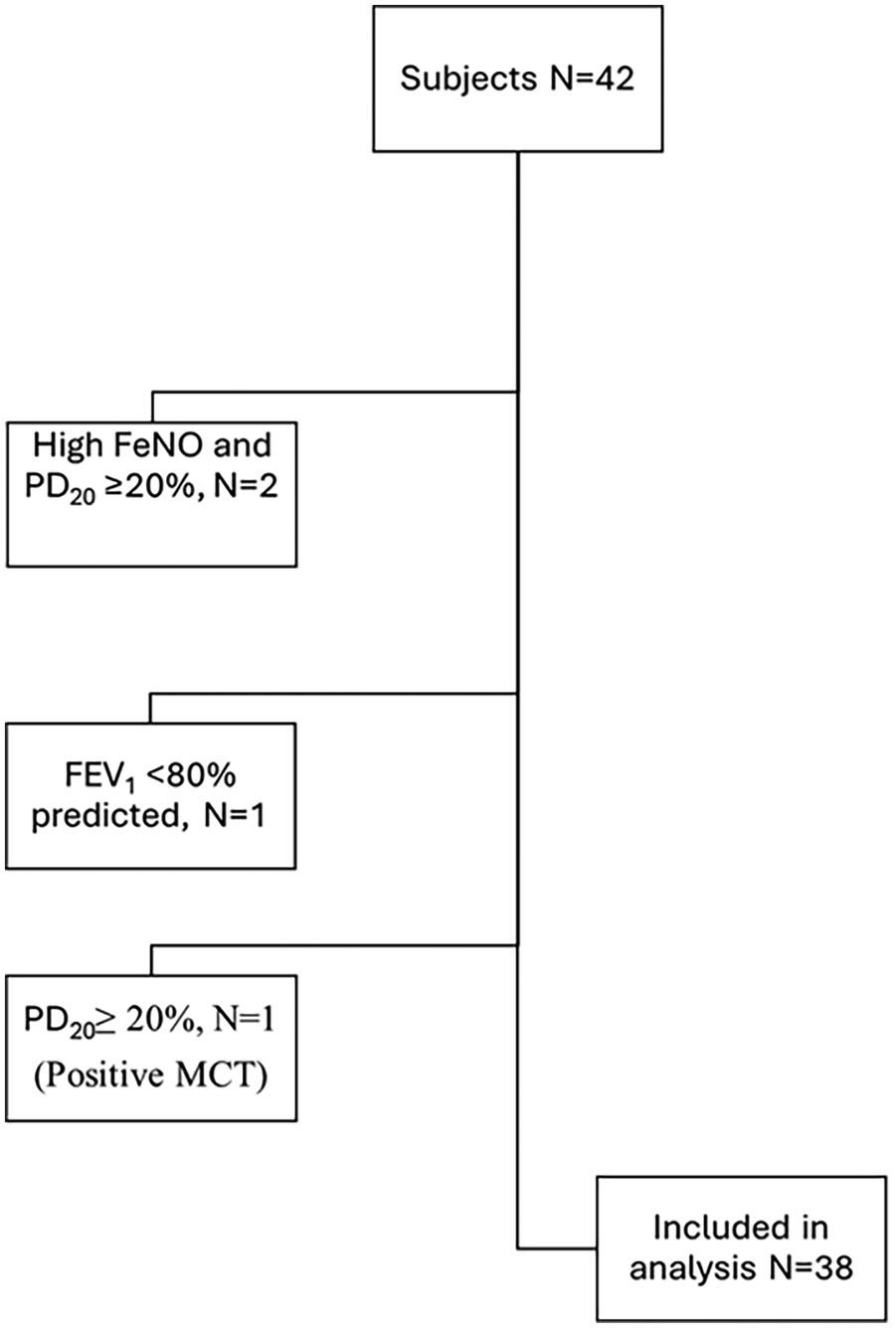
Patient flow diagram. A total of 42 individuals were assessed for eligibility. Four participants were excluded: two due to elevated fractional exhaled nitric oxide (FeNO > 25 ppb) combined with a PD₂₀ ≥ 20%, one due to a baseline FEV₁ < 80% predicted, and one due solely to a positive methacholine challenge (PD₂₀ ≥ 20%) without other exclusion criteria. The remaining 38 participants fulfilled all inclusion criteria and were included in the final analysis.

Adults aged 18–65 years, never-smokers or ex-smokers with <5 pack-years, were screened for eligibility. Key inclusion criteria were normal baseline spirometry (FEV₁ ≥ 80% predicted; FEV₁/FVC >0.70) (24) and FeNO <25 ppb (25). Exclusion criteria included recent respiratory infection, use of inhaled bronchodilators or corticosteroids within 4 weeks, pregnancy or lactation, BMI >40 kg m⁻², or any chronic cardiorespiratory/metabolic disease. Of 42 people screened, 38 met criteria and completed both periods (see CONSORT flow diagram, Figure 1).

### 2.3 Crossover schedule and masking

**Figure 2:**
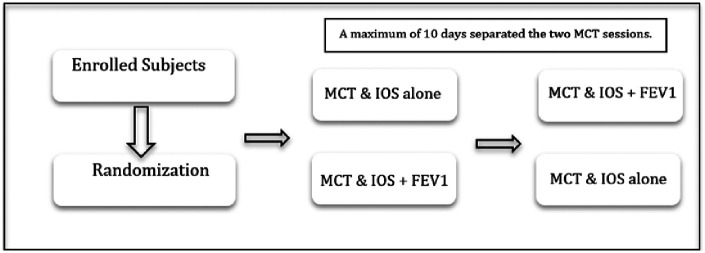
Crossover sequence and test schedule. Each participant completed two methacholine challenge test (MCT) sessions in a quasi-randomized crossover design, separated by no more than 10 days. In Sequence A, participants underwent IOS-only testing at Visit 1 and IOS combined with spirometry (IOS + FEV₁) at Visit 2. In Sequence B, the order was reversed. Spirometry was always performed at least 3 h before MCT 26 to confirm normal lung function and to avoid bronchodilation effects during the subsequent IOS measurement. The order of test sessions was determined by date-of-birth parity (even vs. odd), not by concealed randomization.

Each participant completed two MCT sessions ≤10 days apart: (i) IOS-only at each dose step and (ii) IOS immediately followed by spirometry (IOS + DI) at each dose step. The crossover sequence and testing schedule are illustrated in Figure 2. Sequence allocation used birthdate parity (even vs. odd) to balance order across participants. Operators could not be blinded to protocol; data analysts were masked until database lock.

### 2.4 Methacholine challenge protocol

We used a five-step dosimeter protocol (20 mg mL⁻¹ methacholine; delivered cumulative doses 0.072–1.440 mg), with aerosol delivered using an APS nebulizer calibrated at 0.009 mL·puff⁻¹. Participants breathed tidally for 2 min via a filter mouthpiece. Participants were instructed to maintain stable tidal breathing within the visual feedback range provided by the IOS system (manufacturer-defined “green zone”), corresponding to normal tidal breathing. Although tidal volume was not quantitatively recorded, real-time visual coaching was used to minimize variability in breathing pattern across measurements. IOS recordings (30 s) were acquired 90 s postinhalation; in the IOS + DI arm, spirometry followed immediately after IOS at each dose. Testing ceased at PD₂₀FEV₁ or after dose step 5, consistent with established methacholine challenge testing guidance (27). Salbutamol 400 µg was administered at the end of testing. Participants were monitored for respiratory symptoms throughout testing; however, symptoms were not systematically recorded using a standardized instrument.

### 2.5 Measurements

#### 2.5.1 Impulse oscillometry

Impulse oscillometry was performed using the Vyntus™ IOS system (Vyaire Medical, Germany). Measurements adhered to ERS technical standards (daily 3 L calibration, monthly impedance verification). Three artefact-free recordings with coherence >0.90 at 10 Hz and within-recording variability <10% were required per time point. We analyzed R₅, R₂₀, R₅–R₂₀, X₅, Fres, and AX. The percent-predicted values were derived using established reference equations. Participants were coached to maintain a stable breathing pattern throughout measurements using visual feedback provided by the device. While tidal volume was not recorded for analysis, all recordings were performed under standardized conditions with operator guidance to ensure consistent tidal breathing.

#### 2.5.2 Spirometry and FeNO

Spirometry (ATS/ERS 2019 criteria) (24) included at least three acceptable manoeuvers and up to a maximum of eight attempts per dose step to achieve reproducibility. The potential cumulative effect of repeated deep inspirations on IOS outcomes was not formally modeled; however, all IOS measurements were consistently obtained prior to spirometry at each dose step to minimize immediate bronchodilatory effects. All measurements were performed by trained operators following a standardized protocol, and quality control criteria were applied consistently across all study visits.

### 2.6 Endpoints

#### 2.6.1 Primary endpoint

The main endpoint was within-subject difference in absolute *Δ*R₅ (kPa L⁻¹ s) between IOS-only and IOS + DI at the final common methacholine dose step (1.44 mg).

#### 2.6.2 Secondary endpoints

The secondary endpoints included within-subject percentage changes in R₅, R₅–R₂₀, AX, and Fres across dose steps; PD₄₀R₅ and PD₅₀R₅ thresholds; correlation between baseline R₅ and *Δ*R₅; and protocol-specific ULNs (95th percentile) for each IOS outcome.

### 2.7 Sample size justification

An *a priori* calculation (expected within-subject *Δ*R₅ difference 0.036 kPa L⁻¹ s; SD 0.06) indicated that 32 participants would provide 90% power (*α* = 0.05) in a crossover design (*ρ* = 0.40). We enrolled 38 to allow for attrition; all completed both visits.

### 2.8 Statistical analysis

Continuous variables were screened using the Shapiro–Wilk test; most IOS outcomes were non-Gaussian and are summarized as medians (IQR). The primary comparison (IOS-only vs. IOS + DI) used a linear mixed-effects model with random intercept (participant) and fixed effects for protocol, dose, period, and protocol×dose interaction; carryover was tested via a period×sequence term (two-sided *α* = 0.05). Exact paired Wilcoxon tests provided non-parametric sensitivity analyses. All analyses were conducted on complete -case data, as no missing outcome data were observed. Family-wise error across six prespecified IOS endpoints (R₅%*Δ*, R₅–R₂₀, AX, X₅, Fres, R₅%pred) was controlled using Holm–Bonferroni correction. ULNs were derived from the IOS-only dataset using 10,000 bias-corrected and accelerated (BCa) bootstrap resamples (reporting the empirical 95th percentile and BCa 95% CI). Analyses were performed in SPSS 29.0.2 and R 4.4.0. Two-tailed *p* < 0.05 was considered significant. Externally validated IOS reference equations were not used for defining ULNs, as no universally accepted reference standards exist for methacholine challenge conditions and IOS-derived bronchial responsiveness. Given known device- and population-specific variability, ULNs were derived internally to ensure methodological consistency within the study. Model assumptions (normality and homoscedasticity of residuals) were evaluated by visual inspection of diagnostic plots.

### 2.9 Patient and public involvement

Patients or members of the public were not involved in the design, conduct, reporting, or dissemination of this research.

### 2.10 Ethics

The protocol was approved by the Regional Health Research Ethics Committee of the Capital Region of Denmark (H-25000081). All procedures conformed to the Declaration of Helsinki. Written informed consent was obtained from all participants. The trial registration number is NCT05012345.

## 3 Results

### 3.1 Participant characteristics

Thirty-eight healthy volunteers completed both methacholine challenge sessions (52.6% female; median age 34.8 years, range 18.9–61.5; median BMI 23.5 kg m⁻², range 18.3–36.1). Baseline spirometry and IOS values met reference ranges for all participants (Table 1). No participant reached PD₂₀FEV₁ within the five-step protocol; the mean (±SD) FEV₁ fall was 7.6 ± 4.9%. No participant spontaneously reported clinically relevant respiratory symptoms during methacholine challenge testing.

**Table 1:** Baseline characteristics and impulse oscillometry (IOS) parameters of the study cohort (*n* = 38).

Variable	Mean/median/(min: max)
Age (years)	38.41/34.83/(18.92: 61.51)
Female sex (%)	52.6
Body mass index (kg m⁻²)	23.70/23.50/(18.30: 36.10)
FeNO (ppb)	14.16/14.50/(3.00: 22.00)
SpO₂ (%)	99.0/99.0/(96.0: 100.0)
FEV₁ (% predicted)	100.3/100.0/(80.0: 125.0)
FEV₁/FVC (%)	83.5/83.0/(71.0: 99.0)
R₅ (kPa L⁻¹ s) (visit 1)	0.25/0.23/(0.14: 0.45)
R₅ (kPa L⁻¹ s) (visit 2)	0.25/0.23/(0.15: 0.43)
R₅–R₂₀ (kPa L⁻¹ s) (visit 1)	0.01/0.00/(0.00: 0.07)
R₅–R₂₀ (kPa L⁻¹ s) (visit 2)	0.01/0.00/(0.00: 0.08)
AX (kPa L⁻¹) (visit 1)	0.16/0.15/(0.02: 0.42)
AX (kPa L⁻¹) (visit 2)	0.15/0.13/(0.03: 0.59)
X₅ (kPa L⁻¹ s) (visit 1)	−0.08/−0.08/(−0.14: −0.02)
X₅ (kPa L⁻¹ s) (visit 2)	−0.07/−0.07/(−0.14: −0.03)
Fres (Hz) (visit 1)	9.15/8.74/(6.64: 13.83)
Fres (Hz) (visit 2)	9.10/8.50/(6.69: 16.14)

Continuous data are presented as median (range); categorical variables as percentages. BMI, body mass index; FEV₁, forced expiratory volume in 1 second; FVC, forced vital capacity; FeNO, fractional exhaled nitric oxide; IOS, impulse oscillometry; SpO₂, peripheral oxygen saturation; R₅, total airway resistance at 5 Hz; R₅–R₂₀, frequency dependence of resistance; AX, area under the reactance curve; X_5_, Reactance at 5 Hz; Fres, resonant frequency.

**Table 2:** Comparison of IOS responses with and without DI at the final common methacholine dose (dose 5).

Parameter	IOS alone [Median (Q1–Q3)]	IOS + DI [Median (Q1–Q3)]	Wilcoxon *p*-value
*Δ*R5 (% rel.)	87.0 [45.8–137.8]	60.4 [21.6–86.5]	<0.001
R5 (% pred.)	80.2 [66.9–87.6]	78.0 [64.6–85.7]	0.997
*Δ*R20 (kPa/L/s)	0.07 [0.05–0.12]	0.07 [0.03–0.10]	0.272
*Δ*R5–R20 (kPa/L/s)	0.14 [0.01–0.20]	0.04 [0.00–0.10]	<0.001
*Δ*AX (kPa/L)	1.55 [0.09–3.22]	0.50 [0.07–1.11]	<0.001
*Δ*X5 (kPa/L)	−0.13 [−0.18 to −0.03]	−0.06 [−0.08 to −0.02]	<0.001
*Δ*Fres (Hz)	15.10 [1.54–20.89]	7.27 [1.33–13.33]	<0.001

*Δ*, change from baseline; DI, deep inspiration. Wilcoxon *p*-values are paired within-subject.

Values are presented as median [interquartile range]. *Δ* denotes change from baseline to the highest administered methacholine dose (Dose 5). IOS Alone refers to the protocol without deep inspiration (DI), whereas IOS + FEV₁ includes concurrent spirometry maneuvers involving DI. R5 (% predicted) represents the absolute baseline R5 expressed as a percentage of the predicted normal value. *Δ*R5 (% relative) reflects the relative change in R5 at the final dose, expressed as a percentage of the predicted baseline R5. Wilcoxon signed-rank tests were used for within-subject comparisons of paired outcomes between protocols.

**Table 3:** Methacholine dose steps provoking bronchial reactivity thresholds (*n* = 38).

Metric	IOS-only median dose	IOS + DI median dose	*p*-Value
PD_40_ R_5_	4	4	0.136
PD_50_ R_5_	4	5	0.051
PD_20_ FEV_1_	6	6	—

PD, provocative dose step; DI, deep inspiration.

Values represent median methacholine dose steps (1–5) required to provoke the indicated response. A value of 6 denotes that the threshold was not reached within the five-step protocol. PD = provocative dose; DI = deep inspiration; R₅ = total airway resistance at 5 Hz; FEV₁ = forced expiratory volume in 1 s.

PD₄₀R₅ = first step with ≥40% rise in R₅; PD₅₀R₅ = ≥50% rise in R₅; PD₂₀FEV₁ = ≥20% fall in FEV₁. *p*-values from paired Wilcoxon signed-rank tests.

**Table 4A:** Upper limits of normal (ULN) for IOS responses to MCT.

Parameter	IOS alone	IOS + DI
R5 (% rel.)	179.6	134.4
Fres	30.97	22.92
R5–R20 (kPa/L/s)	0.40	0.32
X5	−0.61	−0.31
AX (kPa/L)	5.58	4.14

ULNs are empirical 95th percentiles (5th for X5). Separate estimates are shown for IOS-only and IOS + deep inspiration (DI).

Unadjusted 95th-percentile ULNs derived directly from the observed distribution of each IOS parameter in the healthy reference group. Values reflect either R5 relative change from baseline (% rel.) or absolute change (for X₅, AX, fres, and R₅–R₂₀), as appropriate. Separate ULNs are reported for the IOS-only protocol and the IOS + spirometry (DI) protocol to illustrate the physiological effect of deep inspiration. These uncorrected values serve as the empirical basis for the statistical bias correction applied in Table 4B. These values are unadjusted for sampling variability and serve as the raw reference from which bias-corrected ULNs in Table 4B were derived.

**Table 4B:** Empirical ULNs with BCa 95% confidence intervals for methacholine-induced IOS changes.

Parameter	IOS alone: ULN	95% CI (lower)	95% CI (upper)	IOS + DI: ULN	95% CI (lower)	95% CI (upper)
R5 (% rel.)	179.62	153.11	183.33	134.39	121.41	140.91
Fres	30.97	26.50	32.65	22.92	16.77	24.08
R5–R20	0.40	0.31	0.42	0.32	0.20	0.35
X5	−0.61	−0.83	−0.45	−0.31	−0.73	−0.24
AX	5.58	4.74	6.54	4.14	2.60	4.39

ULN = 95th percentile (5th for X5). BCa = bias-corrected accelerated bootstrap with 10,000 resamples; 95% CIs are BCa intervals.

The ULN reported for each parameter is the empirical percentile (95th; 5th for X5) from Table 4A; BCa bootstrap (10,000 resamples) was used only to derive confidence intervals. The “IOS-only” dataset was the prespecified normative reference; “IOS + DI” is shown for comparison. With this sample (*n* = 38), bias of the percentile estimator was negligible, so point estimates in Table 4B closely match those in Table 4A.

### 3.2 Primary endpoint: *Δ*r₅ at the final methacholine dose

At Dose 5, the absolute rise in total airway resistance (*Δ*R₅) was larger during IOS-only compared with IOS + DI testing. The linear mixed-effects model (random intercept = participant) estimated an adjusted within-subject difference of +0.074 ± 0.021 kPa L⁻¹ s (t₃₇ = 3.50, *p* = 0.0012). The prespecified sensitivity analysis (exact paired Wilcoxon) corroborated this finding (median +0.07 kPa L⁻¹ s; 95% CI +0.03 to +0.11; *p* = 5.2 × 10⁻⁴). Model diagnostics showed no significant protocol×dose interaction, period effect, or carryover.

### 3.3 Secondary endpoints

Percentage changes showed consistent attenuation with DI. Median increases in R₅, R₅–R₂₀, AX, and Fres were 50%–75% smaller when a DI-requiring spirometry maneuver followed each IOS measurement, whereas central resistance (R₂₀) was protocol-independent (*p* = 0.21).

### 3.4 Provocative dose thresholds

The dose step provoking a ≥ 40% rise in R₅ (PD₄₀R₅) was unchanged between protocols (median = Dose 4; Wilcoxon *p* = 0.136). In contrast, the ≥50% threshold (PD₅₀R₅) occurred one step later with DI (median Dose 5 vs. Dose 4; *p* = 0.051). No participant met PD₂₀FEV₁; by convention this was encoded as 6 (threshold not reached within the maximum cumulative dose). Detailed protocol-specific IOS responses and provocative-dose threshold comparisons are provided in Tables 2, 3.

### 3.5 Dose–response profiles

**Figure 3:**
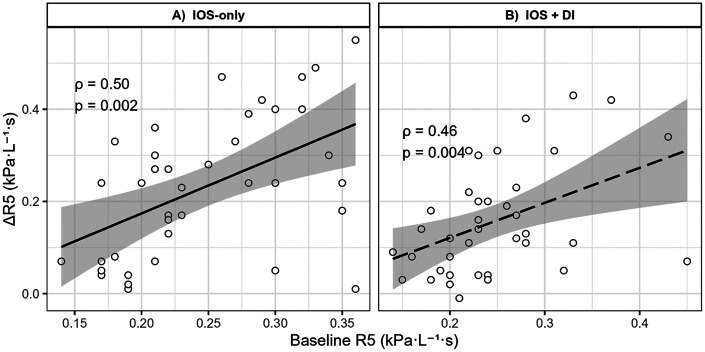
Dose–response profiles of IOS outcomes during methacholine challenge under IOS-only and IOS + DI protocols. Data are shown as median change from baseline (*Δ*) with interquartile range at baseline and dose steps D1–D5. Open circles, solid line = IOS-only; triangles, dashed line = IOS + DI (with deep inspiration). *Y*-axes are scaled independently per panel. Asterisks mark paired Wilcoxon tests between protocols at D3–D5 with Holm correction (*p* < 0.05; *p < 0.01; **p < 0.001). R₅ = total airway resistance; R₅–R₂₀ = frequency dependence of resistance; AX = reactance area; Fres = resonant frequency.

Figure 3 illustrates the progressive divergence between protocols from dose step 3 onward, with consistently greater increases in IOS-only measurements. All IOS indices increased in a dose-dependent manner under both protocols. From Dose 3 onward, responses diverged, with larger changes observed in the IOS-only condition. By Dose 5, median values (IOS-only vs. IOS + DI) were as follows:

- R₅ (*Δ* kPa L⁻¹ s): 0.24 vs. 0.14 (*p* < 0.01)
- R₅–R₂₀ (*Δ* kPa L⁻¹ s): 0.13 vs. 0.04 (*p* < 0.01)
- AX (*Δ* kPa L): 1.55 vs. 0.50 (*p* < 0.001)
- Fres (*Δ* Hz): 15.1 vs. 7.3 (*p* < 0.001)

### 3.6 Upper limits of normal

Empirical 95th-percentile ULNs (and BCa 95% CIs from 10,000 bootstrap resamples) derived from the IOS-only dataset were higher than those observed with DI, consistent with bronchoprotection from deep inspiration. The values were as follows:

- R₅ (% relative): 179.6% (153.1–183.3) vs. 134.4% (121.4–140.9)
- Fres (Hz): 30.97 (26.50–32.65) vs. 22.92 (16.77–24.08)
- R₅–R₂₀ (kPa L⁻¹ s): 0.40 (0.31–0.42) vs. 0.32 (0.20–0.35)
- X₅ (kPa L⁻¹ s; 5th percentile): −0.61 (−0.83 to −0.45) vs. −0.31 (−0.73 to −0.24)
- AX (kPa L): 5.58 (4.74–6.54) vs. 4.14 (2.60–4.39)

### 3.7 Exploratory analyses

**Figure 4:**
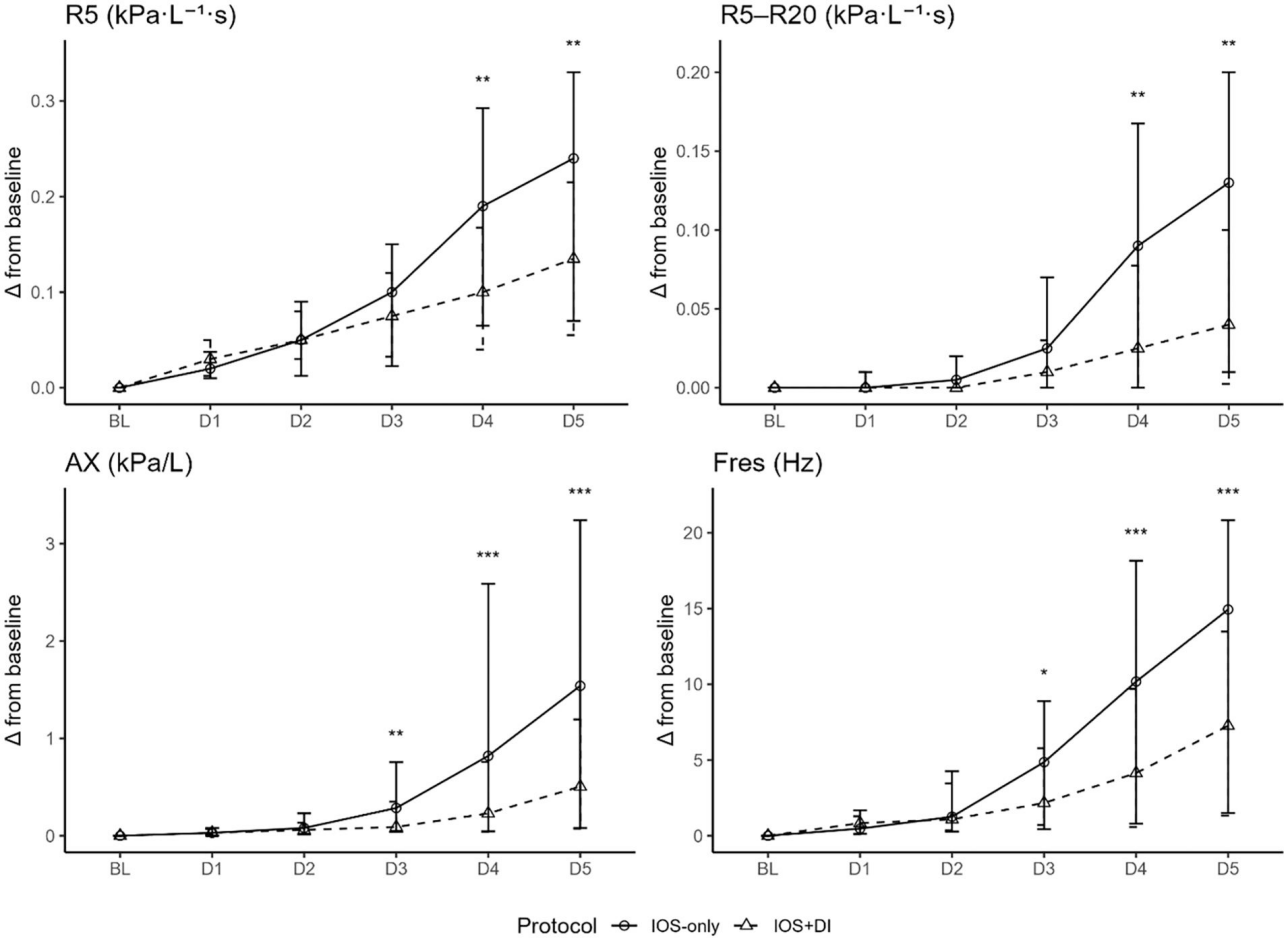
Baseline R₅ versus methacholine-induced change in R₅. The left plot shows the IOS-only protocol, and the right plot shows the IOS + DI protocol. Each dot represents one participant (*n* = 38). Solid line = linear regression with 95% confidence band (gray shading). Statistics shown in each plot are Spearman correlation coefficients (*ρ*) with corresponding *p*-values. In both protocols, higher resting R₅ values were associated with larger absolute increases in R₅ at Dose 5, although responses were attenuated in the IOS + DI condition.

**Figure 5:**
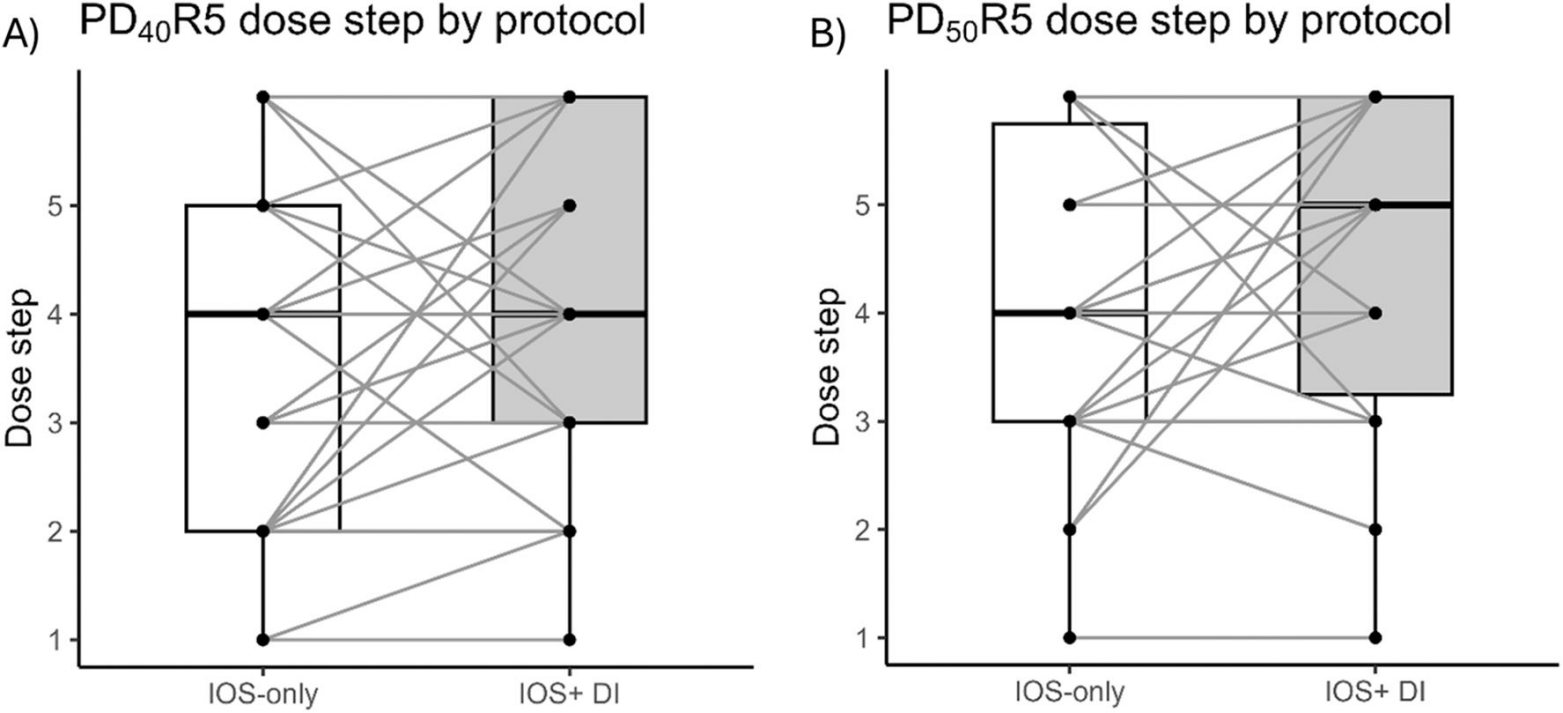
Individual within-subject changes in methacholine dose steps at which bronchial reactivity thresholds were reached under the two protocols. Panel **(A)** shows PD₄₀R₅, defined as the first methacholine dose step producing a ≥40% increase in R₅ from baseline (*n* = 21/38). Panel **(B)** shows PD₅₀R₅, defined as the first step producing a ≥ 50% increase (*n* = 18/38). Only participants who reached the respective threshold in both protocols within the five-step schedule are displayed. Each line connects one participant’s IOS-only result (left) with the corresponding IOS + DI result (right). An upward slope indicates that a higher methacholine dose was required when spirometry/deep inspiration was included, consistent with attenuation of airway responsiveness; a downward slope indicates the opposite. Boxplots summarize the distribution of dose steps in each protocol (**A**: Wilcoxon V = 98, *p* = 0.136; **B**: V = 91, *p* = 0.051).

Baseline FeNO was uniformly low (median 14.5 ppb, IQR 11.5–17.8) and showed no correlation with maximal methacholine-induced changes in any IOS endpoint (all |*ρ*| ≤ 0.20; Holm-adjusted *p* > 0.60; Spearman). In contrast, higher resting R₅ values were positively associated with larger absolute rises in R₅ at the highest cumulative methacholine dose (Dose 5) under both test protocols. In particular, in the IOS-only condition, the correlation between baseline R₅ and *Δ*R₅ was moderate and statistically significant (*ρ* = 0.50, *p* = 0.002); a similar pattern was observed when deep inspiration was included (IOS + DI: *ρ* = 0.46, *p* = 0.004), despite overall attenuation of responses (Figure 4). No other baseline variable (age, BMI, FEV₁ %pred) showed a consistent association. Individual within-subject changes in the methacholine dose steps at which PD₄₀R₅ and PD₅₀R₅ thresholds were reached are shown in Figure 5. Across endpoints, adding a DI-requiring spirometry maneuver was consistently associated with smaller methacholine-induced changes, later provocative dose thresholds, and lower calculated ULNs compared with IOS-only measurements.

## 4 Discussion

### 4.1 Principal findings

In a rigorously screened cohort of healthy adults undergoing a standard five-dose MCT, a forced DI associated with spirometry substantially attenuated methacholine-induced changes in IOS indices reflecting small-airway mechanics. The effect was consistent across R₅, R₅–R₂₀, AX, and Fres, emerged from dose step 3 onward, and delayed PD₅₀R₅ by one full step, while PD₂₀FEV₁ was not reached in any participant. Bootstrap-derived, protocol-specific ULNs were higher in the IOS-only condition, underscoring that DI materially shifts interpretive thresholds. These findings support guidance to obtain IOS before any forced maneuver during bronchial challenge and demonstrate that protocol choice (IOS-only vs. IOS + DI) is not interchangeable for diagnostic interpretation.

### 4.2 Context with prior work

Only two earlier studies have directly explored the impact of DI on methacholine IOS responses, both in small mixed cohorts (14, 17). Remarkably, only one prior study has explored how simultaneous spirometry affects IOS measurements during a standard methacholine challenge test (14). In a study of 16 asthmatic patients, DI required for spirometry to track lung function during an MCT attenuated the IOS response: On average R5, R5-20, and AX were 55.9%, 57.5%, and 65% greater, respectively, in the challenge without than with spirometry (14). Our data replicate and extend those observations in a rigorously screened, normal-weight, healthy population using a strictly standardized dosimeter method. In addition, the present dose-by-dose analysis confirms a progressive divergence between protocols, indicating cumulative bronchoprotection rather than a transient dilatory response.

Based on the observed correlation between a 20% decrease in FEV₁ and a 40% increase in R₅ during MCT, IOS device manufacturers have proposed a 40% rise in R₅ as the threshold above which an IOS response would be considered abnormal in a clinical context (11). We show that healthy adults frequently exceed that threshold even when DI is imposed, whereas IOS-only testing yields median R₅ increases of ∼90%, suggesting that a universal 40%–50% cutoff is overly sensitive and protocol-dependent. Our findings are consistent with earlier small-scale studies in healthy adults, where R₅ increased by 123% and 133%, respectively, following MCT without concurrent spirometry (15, 17). Conversely, studies incorporating simultaneous spirometry have reported more modest increases in R₅, ranging from 40% to 70% (12, 13, 16, 17).

### 4.3 Mechanistic considerations

A deep inspiration acutely stretches airway smooth muscle and transiently increases lung volume, producing bronchodilation and a bronchoprotective effect that blunts subsequent constriction; this protection is prominent in healthy airways and attenuated or absent in asthma (19–22, 26). In our design, IOS was acquired before each DI in the IOS + DI arm, so the smaller IOS changes are unlikely to be explained by immediate post-DI dilation. Rather, the preceding DI at the prior dose step likely reduced responsiveness at the next increment, consistent with strain adaptation of airway smooth muscle and cumulative protection over successive steps.

### 4.4 Clinical implications

When spirometry is impracticable or contraindicated, IOS-only MCT offers a sensitive, effort-independent option for detecting early peripheral airway reactivity (29–31). However, interpretation must reference the acquisition protocol: Applying IOS-only ULNs to data collected after DI risks misclassification (typically under-calling abnormality because DI suppresses *Δ*R₅–R₂₀, *Δ*AX, and *Δ*Fres by 2- to 3-fold on average). The observed correlation between baseline R₅ and absolute *Δ*R₅ (*ρ* ≈ 0.5) suggests that resting peripheral resistance can help anticipate the magnitude of provoked change, whereas FeNO, age, and BMI did not add explanatory value in this healthy cohort (31). Together, these points argue for protocol-specific reporting and adopting data-derived ULNs rather than universal manufacturer thresholds. Importantly, the absence of standardized IOS thresholds and externally validated reference equations for bronchial challenge testing limits direct clinical translation. Our internally derived ULNs therefore represent protocol-specific reference values rather than universally applicable diagnostic cutoffs.

### 4.5 Strengths and limitations

Strengths include the prospective crossover design, tight procedural control of DI, a standardized five-dose dosimeter MCT, rigorous screening to exclude latent hyperresponsiveness, and non-parametric exact tests with Holm correction alongside mixed-effects modeling. We also report bootstrap BCa CIs for ULNs to quantify sampling uncertainty. Limitations include the single-center setting, modest sample size (albeit exceeding typical pilot normative recommendations), and restriction to healthy, predominantly normal-weight adults; results may not generalize to children, older adults, or individuals with obesity or asthma. We did not measure the isolated acute bronchodilator effect of a standardized DI immediately pre-/post-IOS within the same dose step. Future protocols that record IOS before and after a controlled DI at each step could disentangle protection from dilation. We did not record tidal volume during IOS measurements, which may influence oscillometry outcomes. However, all participants were coached to maintain stable tidal breathing using real-time visual feedback, reducing variability in breathing pattern. Future studies should incorporate quantitative tidal volume monitoring to further refine IOS interpretation.

Respiratory symptoms were not systematically assessed using a standardized instrument during MCT, limiting evaluation of the relationship between IOS-derived changes and clinical symptom perception.

In addition, repeated spirometry manoeuvers may exert cumulative bronchoprotective effects via deep inspiration. Although IOS was consistently performed prior to spirometry at each dose step, the potential cumulative impact of repeated deep inspirations across the protocol cannot be fully excluded.

### 4.6 Future directions

Validation of protocol-specific ULNs is warranted in broader populations and across devices, with attention to age, obesity, and asthma phenotypes where DI effects may differ (28). Integrating IOS-based reactivity with multiomics and clinical phenotyping could clarify mechanisms that govern interindividual variation in DI responsiveness and support refined endotype-aware thresholds for small-airway dysfunction (29–31).

## 5 Conclusions

Deep inspiration associated with spirometry materially attenuates methacholine-induced IOS responses—most notably small-airway indices (R₅–R₂₀, AX, and Fres)—from mid-dose onward, delays PD₅₀R₅, and prevents PD₂₀FEV₁ in rigorously screened healthy adults. Interpretation of bronchial challenges using IOS must therefore be protocol-specific: Thresholds derived from IOS-only testing should not be applied to data collected after DI. Our bootstrap-derived, protocol-specific ULNs offer practical, data-driven reference values to support clinical use when spirometry is impracticable. These findings support protocol-specific interpretation of oscillometry during bronchial challenge testing and may improve diagnostic accuracy when spirometry is impracticable.

## Data Availability

De-identified participant data supporting the conclusions of this article, together with relevant analysis code and metadata, will be made available by the corresponding author upon reasonable request, subject to applicable ethical and institutional approvals.
